# A Smart Visual Sensor for Smoke Detection Based on Deep Neural Networks

**DOI:** 10.3390/s24144519

**Published:** 2024-07-12

**Authors:** Vincenzo Carletti, Antonio Greco, Alessia Saggese, Bruno Vento

**Affiliations:** 1 Department of Information and Electrical Engineering and Applied Mathematics (DIEM), University of Salerno, 84084 Fisciano, Italy; vcarletti@unisa.it (V.C.); asaggese@unisa.it (A.S.); 2 Department of Electrical Engineering and Information Technology (DIETI), University of Napoli Federico II, 80138 Napoli, Italy; bruno.vento@unina.it

**Keywords:** smoke detection, early fire detection

## Abstract

The automatic detection of smoke by analyzing the video stream acquired by traditional surveillance cameras is becoming a more and more interesting problem for the scientific community thanks to the necessity to prevent fires at the very early stages. The adoption of a smart visual sensor, namely a computer vision algorithm running in real time, allows one to overcome the limitations of standard physical sensors. Nevertheless, this is a very challenging problem, due to the strong similarity of the smoke with other environmental elements like clouds, fog and dust. In addition to this challenge, data available for training deep neural networks is limited and not fully representative of real environments. Within this context, in this paper we propose a new method for smoke detection based on the combination of motion and appearance analysis with a modern convolutional neural network (CNN). Moreover, we propose a new dataset, called the MIVIA Smoke Detection Dataset (MIVIA-SDD), publicly available for research purposes; it consists of 129 videos covering about 28 h of recordings. The proposed hybrid method, trained and evaluated on the proposed dataset, demonstrated to be very effective by achieving a 94% smoke recognition rate and, at the same time, a substantially lower false positive rate if compared with fully deep learning-based approaches (14% vs. 100%). Therefore, the proposed combination of motion and appearance analysis with deep learning CNNs can be further investigated to improve the precision of fire detection approaches.

## 1. Introduction

Forest fires are becoming more frequent and fierce as highlighted in the EFFIS (European Forest Fire Information System) Annual Fire Reports [1]. Fires can cause enormous damage in terms of humans and animals deaths and loss of private property and public infrastructure [2]; for example, every year more than three thousand people are killed by fires in the US [3]. Human-based surveillance is not sufficient to guarantee a constant monitoring of the whole forested area of a country; therefore, the availability of effective and accurate systems to detect fires at the early stages is crucial to allow for prompt management, thus reducing the impact of a big fire. To this aim, smoke detection systems play a crucial role. Indeed, when the fire is not yet visible, the smoke generated in the preliminary phase of combustion can be detected even from long distances.

The detection of smoke is usually approached in two different ways [4]: (1) using physical sensors that measure particles, temperature and relative humidity [5,6,7,8] or (2) performing analysis on videos and images acquired by surveillance cameras (visual sensors) [9,10,11,12,13,14,15,16,17,18,19,20,21,22,23,24]. While sensors are commonly used to monitor small and limited areas like rooms, their use is unpractical for monitoring wide areas, such as high warehouses, or outdoor environments, such as landfill or forests. Indeed, in the first case, even if indoor, the number of sensors would be very high for guaranteeing full coverage; moreover, due to the position of the sensor on the ceiling, the alarm would be generated only where the fire has already reached large dimensions. Additionally, in outdoor environments, it is very difficult to install the sensors (for example, in a forest), so then the usage of cameras becomes the only viable solution. This is why in the last years we assisted the growing interest of the scientific community by designing accurate video analytic algorithms able to automatically detect the presence of smoke in real time using visual sensors-based computer vision technologies [25].

**Figure 1:**
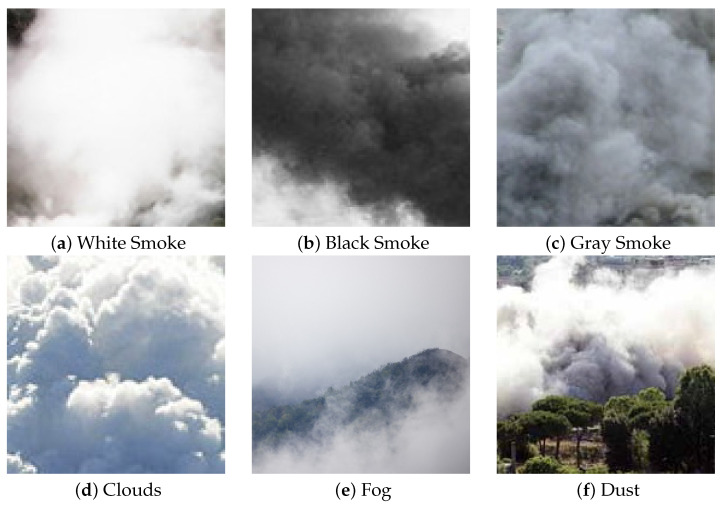
Examples of challenges in smoke detection, namely different colors and similarity to clouds, fog and dust.

There are several factors that make automatic smoke detection through computer vision a very challenging task. The smoke may assume different shades of white, gray and black, and it may not have salient features (for example, it can be dense or sparse). Also, when the area to be monitored is outdoor, it can be easily confused with other elements such as fog or clouds, as evident in Figure 1; the contours of the smoke are very blurred and not highlighted compared to other objects.

For a long time before the advent of deep learning, the literature focused on methods based on handcrafted features, combined with different types of classifiers, such as SVM, KNN, Random Forest or neural networks. Some of the most common features used in the literature are based on motion [9,10,11]. In particular, in [9] the authors propose a method to find potential smoke regions through the Maximally Stable Extremal Region (MSER) detection algorithm. Once the potential smoke region is detected, it is tracked in the subsequent frames. At the same time, the motion vectors of the potential smoke regions are monitored to identify the distinctive upward movement and the expansion of the smoke. Differently, in [11] a cumulative motion model based on the integral image is proposed to quickly estimate the motion orientation of smoke. The collection of the motion vectors over the time is performed to compensate for the errors in the estimation of orientation. In [10], the authors use a condensed image, where static objects are horizontal lines, while shaking objects appear as dithering horizontal lines, in order to find smoke based on the uprising and uniform motion.

**Figure 2:**
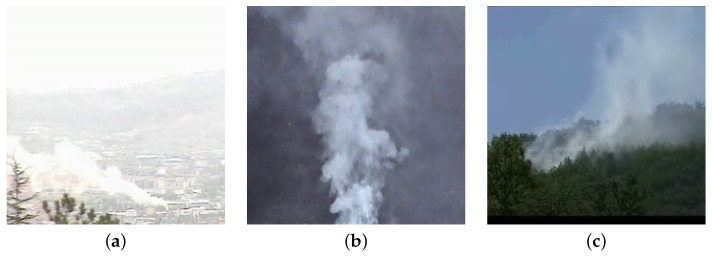
Examples of smoke moving in different directions, namely, left (**a**), upward (**b**) and right (**c**).

In general, motion-based methods assume that the smoke moves only upward; unfortunately this hypothesis does not fit all the cases occurring in the reality, since the presence of adverse weather conditions (such as rain or wind) cause a variation in the typical smoke movement properties, as shown in Figure 2. This causes a drop in accuracy, making these approaches not sufficiently robust when applied in the wild.

Together with motion, other typical features adopted to increase the robustness are color [12,13], texture [14,15,16,17], energy and flickering [18].

In recent years, the disruptive accuracy achieved by convolutional neural networks (CNNs) on image analysis tasks has encouraged the proposal of deep learning-based approaches for smoke detection too [19,20,21,22,23,24]. In particular, in [19] the authors propose the first CNN for smoke recognition that operates directly on the raw RGB frame without the need of the feature extraction stage. A slightly modified version of AlexNet [26] is proposed in [20], to classify smoke against background. In [27], a custom CNN for classifying the entire image is employed. In [22], a joint detection framework based on Faster-RCNN for smoke localization and on a 3D CNN for smoke recognition is proposed. The authors have experimentally demonstrated that replacing the softmax layer with an SVM classifier significantly improves the performance of the system when the amount of training data is quite limited. A deep smoke segmentation network to infer high-quality segmentation masks from blurry smoke images is proposed in [24]. To overcome large variations in the texture, color and shape of smoke appearance, the network is divided into a coarse path and a fine path, which are two encoders-decoder FCNs with skip structures; the output of the two paths is then supplied as input to small networks providing the final outcome.

In [28,29] the authors formalize smoke recognition as a detection problem, by proposing neural networks that jointly locate and classify the smoke. Different typologies of detectors have been used, ranging from Yolov5 [30] to Detectron2 [31], and more recently to transformers [32].

However, it is quite complex, also for humans, to distinguish smoke from dust, fog, clouds or other similar environmental phenomena only looking at the appearance (color, shape or texture) on a single frame without taking into account the movement or the context (see Figure 1). For this reason, other recent papers propose to combine motion and appearance to improve the overall accuracy. In [23], the authors propose to combine two deep neural networks: the first to extract image-based features (smoke color, texture and sharp-edge detection), while the second is specialized on motion-based features (moving region of smoke, growing region and rising region detection); thus, the outcome of the two networks is then combined by an SVM. Differently, Aslan et al. [33] propose the use of a Deep Convolutional Generative Adversarial Network (DCGAN) trained in two stages to obtain a robust representation of sequences with and without smoke. In particular, the learning procedure includes the regular training of a DCGAN with real images and noise vectors, while the temporal evolution of smoke is taken into account through a pre-processing step that applies a motion-based transformation of the images. Finally, in [34] the authors propose a complex architecture that combines deep convolutional recurrent motion–space networks (RMSNs) and Recurrent Neural Networks (RNNs). The RMSNs are used to analyze motion and space context through a very basic CNN architecture, composed of six convolutional layers and a fully connected final stage; the outcome is then combined to feed the RNNs. In the classification stage, a temporal pooling layer that provides a summarized representation of the video sequence is the input of a softmax that predicts the presence of the smoke.

Although the approaches completely based on CNNs are able to achieve remarkable performance on the datasets on which they are trained and evaluated, they have a limited generalization capability in real-world scenarios. One of the reasons is the lack of datasets containing samples representative of all the situations that a smoke detection system is expected to address in the wild; this is true both for popular datasets like [35,36,37], that are based on video collected from real environments, and for those that are composed of synthetic samples [21,38]. Another reason is the complexity of the proposed models that combine motion and appearance; since CNNs can be easily found pre-trained, they can be effectively fine-tuned on new image analysis tasks through transfer learning using few samples. Completely new deep networks, especially if they have a complex architecture with thousands of parameters, require being trained from scratch on a huge amount of samples to avoid overfitting; they are typically specialized on the dataset used for the training and have limited capability to generalize on unseen situations. Therefore, considering the current context, it is clear that the proposal of new datasets is surely needed to extend the scenarios to be used to train such methods. Moreover, there is also the need to work with motion and appearance using more simple approaches that are able to inherit all the benefits of CNNs without adding too much complexity.

According to these considerations, in this paper we provide three main contributions to the state of the art: (i) the proposal of a hybrid method that combines the benefits of traditional motion-based and appearance-based approaches with the modern CNNs; (ii) a new dataset, namely the MIVIA Smoke Detection Dataset (MIVIA-SDD) that contains videos acquired in real environments; (iii) an experimental comparison over the MIVIA-SDD of well-known state-of-the-art CNNs (MobileNet [39], VGG-19 [40], ResNet-50 [41], Inception v3 [42] and Xception [43]). The proposed method firstly analyzes motion and appearance to identify promising regions of the image where the smoke is supposed to be; then, such regions are processed by a CNN to confirm the presence of smoke. The outcome of the CNN is not directly related to an alarm, but different patches are analyzed over time before notifying an alarm to the presence of smoke. This process allows it to achieve a high smoke recognition rate, but at the same time it significantly reduces the false positive rate.

The method has been tested and validated on the MIVIA-SDD, which we made publicly available, composed of 129 videos (59 positive and 70 negative), with about 28 h of videos acquired in real environments. In Table 1, we show the advantages of our dataset with respect to the others, in terms of number and duration of smoke videos. If we analyze the number of frames, we can note that our dataset is approximately 20 times larger than the datasets currently available in the scientific literature. In the experiments, we demonstrate that despite how using only CNNs does not provide a reliable smoke detection system, the addition of a proper pre-processing stage is able to achieve the same accuracy claimed by more complex state-of-the-art approaches.

The paper is organized as follows. In Section 2 we present and discuss the proposed method. The dataset MIVIA-SDD is then described in Section 3, with the addition of some visual examples. In Section 4 we detail the experimental setup and the results. Finally, in Section 5, we draw conclusions and propose further improvements.

## 2. Proposed Method

**Figure 3:**
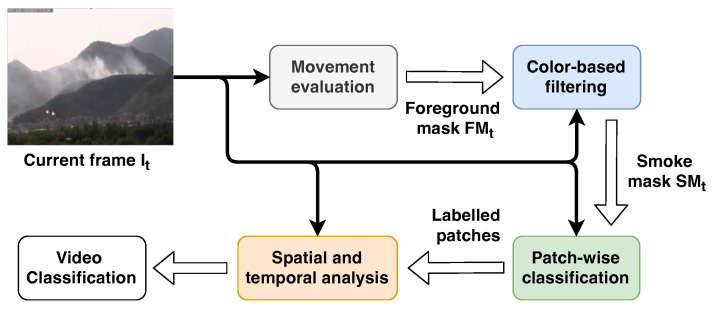
Overview of the proposed method. The current frame ($I_{t}$) is spread to all the stages together with the outcome of each of them. The movement evaluation takes the current frame as input and produces an updated foreground mask $FM_{t}$ to the color-based filtering module, which performs a further refinement of the binary mask by taking into account the appearance. The output of the latter is a binary mask, namely the smoke mask $SM_{t}$, used by the patch-wise classification stage to select the region of the image in which the smoke is expected to be. Finally, the outcome of the classification is provided to the last stage, spatial and temporal analysis, to evaluate the evolution of the smoke over the time and classify the video.

As introduced in Section 1, we propose a hybrid method combining traditional approaches, based mostly on the analysis of movement and color, and CNNs, with the addition of a video-wise evaluation. With this approach, we obtain an accurate detector that is robust in the most common environmental phenomena where fog, cloud and dust can be easily mistaken for smoke. An overview of the proposed method is shown in Figure 3.

The first stage is the movement analysis, which is necessary to distinguish the smoke from the environment under the hypothesis that the former is the only moving object in the scene. This task is commonly performed through the evaluation of a background model representing the environment, updated frame by frame, that is subtracted from the frame under analysis so as to obtain only the pixels belonging to the moving objects, namely the foreground. Obviously, in most of the cases there could be other objects in movement like trees, animals and so on; therefore, we would like to pay attention only on the movement of the smoke. For this reason we use the color to provide a further refinement of the foreground by removing the pixels that do not respect the expected color range. The final foreground image is then converted into a binary image, named smoke mask, which is used to identify the promising regions to feed the CNN. The original frame is then divided in contiguous rectangular regions of 32×32 pixels named patches; only those patches containing more than a given percentage of smoke candidate pixels are provided to the CNN to perform the patch-wise classification. This step determines whether the patch contains the smoke or not. Finally, the current frame is considered to contain smoke if at least one patch is classified as smoke by the CNN. In addition, since the smoke is expected to be not only in the current frame, but in a sequence of frames, a video classifier analyzes the label of a sequence of frames within a time window; if the number of frames containing smoke is higher than a given threshold, the video will be classified as a video with smoke.

According to the previous description, the overall smoke detection process consists of four stages: movement analysis, color-based filtering, patch-wise classification and spatial and temporal analysis. In the next sections we describe each of them in more detail in more detail; the values of the parameters used in our experiments and thoroughly described in the following are reported in Table 2.

### 2.1. Movement Analysis

**Figure 4:**
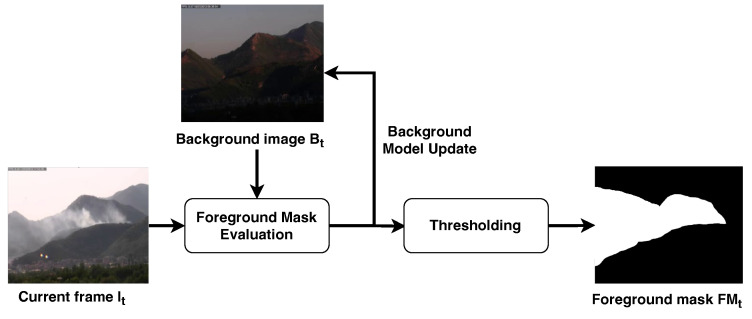
Movement analysis stage. The current frame $I_{t}$ and background image $B_{t}$ are used to update the background image and compute the foreground image $F_{t}$. The latter is then converted to a binary image through thresholding.

The analysis of moving objects, realized with the pipeline shown in Figure 4, is performed through background subtraction [44], which is an approach that is commonly used in video surveillance applications to separate the pixels belonging to objects of interest in the scene from those that are considered static and, thus, part of the environment.

The first step of background subtraction is to build a background model to identify the pixels belonging to the background image, which we denote as $B_{t}$; note that $B_{t}$ is related to the discrete time instant *t* because it is not static, but it evolves over time and has to be updated frame-by-frame. To this purpose, in [45] the authors propose using a model based on a mobile weighted average reported in the following:

(1) $$B_{t}\left(x,y\right)=\alpha_{t}\times I_{t}\left(x,y\right)+\left(1-\alpha_{t}\right)\times B_{t-1}\left(x,y\right)$$

The background image at the current discrete time instant *t* is updated by taking into account both the current frame $I_{t}$ and the background image $B_{t-1}$ computed at the previous discrete time instant t−1. Note that the update is performed pixel-by-pixel as highlighted by the notation $B_{t}\left(x,y\right)$, which refers to the pixel at the coordinate (x,y) of the background image. The two terms $I_{t}\left(x,y\right)$ and $B_{t-1}\left(x,y\right)$ are weighted differently through factor $\alpha_{t}$ that is also updated frame-by-frame according to the following rule:

(2) $$\alpha_{t}=1-e^{-\frac{\delta_{t}}{T}}$$

The aim of such a time-dependent weight is to gradually incorporate a pixel in the background image as it remains static over time. The time required to absorb a pixel into the background is regulated by the term $\delta_{t}$, namely the inter-frame time, which represents the time between two consecutive frames, together with the constant *T*, which approximates the maximum time to be waited before considering the pixel static.

Once the background image $B_{t}$ has been obtained, we can compute the foreground image $F_{t}$ as the absolute difference between the current frame $I_{t}$ and $B_{t}$.

(3) $$F_{t}\left(x,y\right)=\left|I_{t}\left(x,y\right)-B_{t}\left(x,y\right)\right|$$

In particular, observing the evolution of the image $F_{t}$ over time, the effect obtained is that a static pixel vanishes from the foreground image in a time *T*. To this aim, a binary mask $FM_{t}$, named foreground mask, is built by thresholding $F_{t}$ as follows:

(4) $$FM_{t}\left(x,y\right)=\left\{\begin{array}{c} 1,\;\mathrm{if}\;F_{t}\left(x,y\right)>\tau_{F}\\ 0,\;\mathrm{otherwise} \end{array}\right.$$

The effect of such a threshold is to consider as moving pixels only the ones whose intensity value are higher than the threshold $\tau_{F}$. It is common to remove from $FM_{t}$ the pixels that are going to vanish from $F_{t}$, since the difference between that pixels in the current frame $I_{t}$ and in the background $B_{t}$ is not significant.

### 2.2. Color-Based Filtering

Color is as relevant as the movement to distinguish smoke from environmental phenomena. In fact, it can assume different colors and saturation on the base of the combustible material that is burning; therefore, it is important to have the possibility to manually set filtering criteria according to the smoke we are interested in detecting. In [46], the authors discuss a criterion to set the threshold of a color filter. Indeed, they have experimentally demonstrated that the RGB components of the smoke pixels are very close each other, independently from the specific shades of white, gray and black. Given a generic frame $I_{t}$ at the time instant *t* and where $I_{t}^{R}\left(x,y\right)$, $I_{t}^{G}\left(x,y\right)$ and $I_{t}^{B}\left(x,y\right)$ are the three components of the image on the Red, Green and Blue channels, we compute the color mask $C_{t}$ of the frame $I_{t}$ as formalized in Equation (5), by defining a single threshold $\tau_{C}$ configurable by the user in the range [0, 255].

(5) $$C_{t}\left(x,y\right)=\left\{\begin{array}{c} 1,\;\mathrm{if}\;|I_{t}^{B}\left(x,y\right)-I_{t}^{G}\left(x,y\right)|<\tau_{C}\\ \;\;\wedge \;|I_{t}^{G}\left(x,y\right)-I_{t}^{R}\left(x,y\right)|<\tau_{C}\\ \;\;\wedge \;|I_{t}^{R}\left(x,y\right)-I_{t}^{B}\left(x,y\right)|<\tau_{C}\\ 0,\;\mathrm{otherwise} \end{array}\right.$$

Working on the RGB components is not the only criterion that can be adopted to detect the presence of smoke in the scene. In a recent survey on video smoke detection [47], it is highlighted how the presence of smoke can substantially affect the saturation and the brightness of the objects that are behind it. According to this observation, we also apply a saturation thresholding over the image in the color filtering stage. With $I_{t}^{S}$ as the saturation component and $I_{t}^{V}$ as the value component of the frame $I_{t}$ in the HSV color space, we compute the saturation mask $S_{t}$ as shown in Equation (6), by using $\tau_{S}$ and $\tau_{V}$ as saturation and value thresholds, respectively.

(6) $$S_{t}\left(x,y\right)=\left\{\begin{array}{c} 1,\;\mathrm{if}\;I_{t}^{S}\left(x,y\right)<\tau_{S}\;\wedge \;I_{t}^{V}\left(x,y\right)<\tau_{V}\\ 0,\;\mathrm{otherwise} \end{array}\right.$$

**Figure 5:**
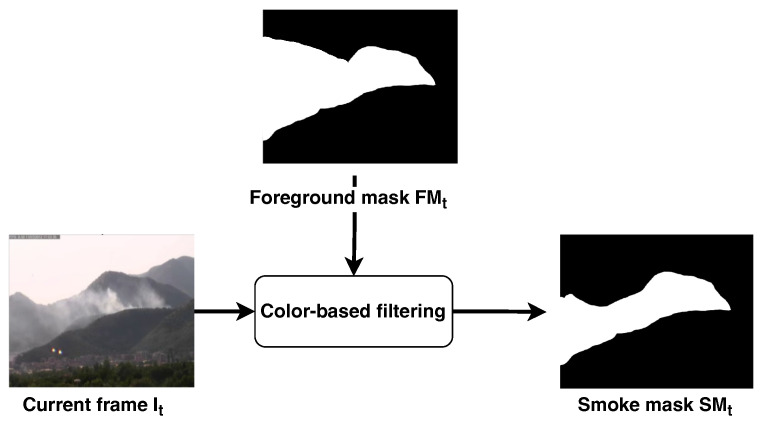
Color filtering step. The foreground mask $FM_{t}$ computed during the movement analysis is used to highlight the area of the current frame $I_{t}$ that has to be processed by the color filter. The output of this stage is a refined binary mask, namely the smoke mask, where the color criteria discussed in Section 2.2 have been applied.

The smoke mask $SM_{t}$ of the frame $I_{t}$ is the combination of the foreground mask $FM_{t}$ computed in the motion analysis, the color mask $C_{t}$ and the saturation mask $S_{t}$, as shown in Figure 5. The resulting smoke mask is used in the subsequent stages to select the regions of interest to be processed by the CNN:

(7) $$SM_{t}\left(x,y\right)=\left\{\begin{array}{c} 1,\;\mathrm{if}\;C_{t}\left(x,y\right)=1\;\wedge \;S_{t}\left(x,y\right)=1\;\wedge \;FM_{t}\left(x,y\right)=1\\ 0,\;\mathrm{otherwise} \end{array}\right.$$

### 2.3. Patch-Wise Classification

Smoke does not have a sharp outline [48]. Therefore, if we select the objects to be classified by the CNN using approaches based on connected component labeling that are widely adopted for the detection of objects of interest on a binary mask [47], we will obtain bounding boxes with wide variability in size and aspect ratio. This choice would complicate the training of CNNs, which have a fixed input image size, because we have to re-scale the image before providing it to classifier. To the best of our knowledge, this problem is not properly addressed in the literature.

**Figure 6:**
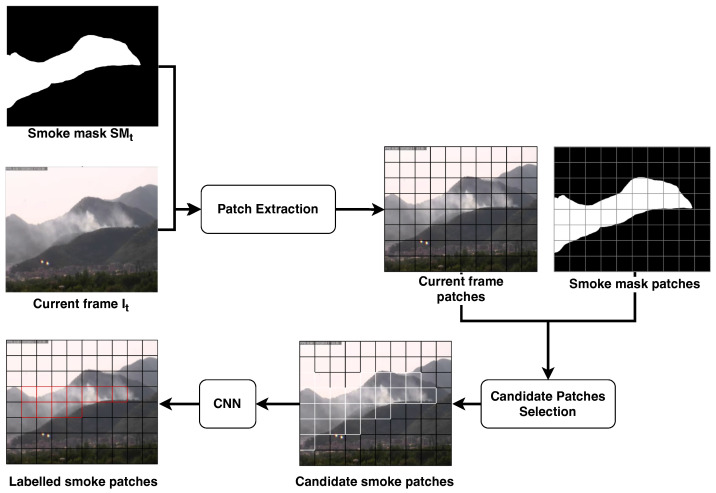
Outline of the patch-wise classification described in Section 2.3. The current frame $I_{t}$ and the smoke mask $SM_{t}$ are both divided in patches of K×K pixels; during the candidate selection, only the patches containing a percentage of white pixels higher than $\tau_{NZ}$ are provided to the CNN to be confirmed as smoke patches. The output of this stage is the set of all the patches with the corresponding label: smoke or no-smoke.

In this paper, we propose to solve this problem by dividing the image through a grid, as shown in Figure 6. Therefore, we obtain a set of non-overlapped adjacent sub-images of the K×K pixels area, namely patches. In this way, we can train and apply the smoke classifier on image patches that always have the same aspect ratio and we can dynamically adapt the size *K* of the patches according to the distance from the smoke. In more detail, the patch division is applied both on the current smoke mask $SM_{t}$ and on the current frame $I_{t}$; each patch is identified by the coordinate over the image of its top-left pixel (i,j), in order to map directly a patch $SM_{t}\left(i,j\right)$ on the smoke mask with the corresponding one on the current frame, namely $I_{t}\left(i,j\right)$.

The patch division is applied over the smoke mask $SM_{t}$ first, in order to evaluate the patches that may contain smoke according to the motion and color filtering. The purpose is to select from the smoke mask the patches having a percentage of foreground pixels higher than a given threshold $\tau_{NZ}$, thus extract the corresponding patches from the current frame and provide them to the patch classifier. In Equation (8), we compute the percentage $W(SM_{t}\left(i,j\right))$ of non-zero pixels with respect to the total number of pixels in the patch $SM_{t}\left(i,j\right)$.

(8) $$W\left(SM_{t}\left(i,j\right)\right)=\frac{\sum_{\left(x,y\right)\in SM_{t}\left(i,j\right)}SM_{t}\left(i,j\right)\left(x,y\right)}{K\times K}$$

For each patch (i,j) we evaluate if the percentage $W(SM_{t}\left(i,j\right))$ is higher than a given threshold $\tau_{NZ}$:

(9) $$P_{t}\left(i,j\right)=\left\{\begin{array}{c} 1,\;\mathrm{if}\;W\left(SM_{t}\left(i,j\right)\right)>\tau_{NZ}\\ 0,\;\mathrm{otherwise} \end{array}\right.$$

If the patch is not promising ($P_{t}\left(i,j\right)=0$), it is automatically classified as background and then no further investigations are required; vice-versa, if ($P_{t}\left(i,j\right)=1$), the corresponding patch $I_{t}\left(i,j\right)$ of the current frame is provided as input to the smoke classifier. It is important to point out that this preliminary filtering of the promising patches allows one to reduce the number of classifications required for each frame and, consequently, to speed up the overall frame processing.

As discussed in Section 1, in this paper we experimentally compared the following state-of-the-art convolutional neural networks to perform patch classification: MobileNetV2 [39], VGG-19 [40], ResNet-50 [41], Inception v3 [42] and Xception [43]. Details about the experimental results are reported in Section 4.

### 2.4. Spatial and Temporal Analysis

**Figure 7:**
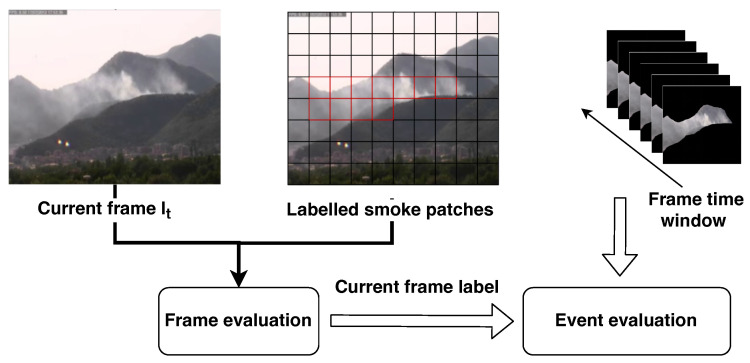
Overview of the spatial and temporal analysis stage discussed in Section 2.4. The current frame $I_{t}$ together with its classified patches are analyzed to classify the entire frame $I_{t}$. The output of the frame-based classification is provided to the video classifier that collects in an internal buffer a set of consecutive frames contained in a configured time window. If more than x% of consecutive frames are classified as smoke, the whole time window is considered to contain smoke and an alarm is raised.

Although the classifier is able to guarantee a satisfying accuracy on the analysis of the patches, having a few of them classified as smoke is not sufficient to fire an alarm. Indeed, we will demonstrate in Section 4.2 that even with the most accurate classifier it is not possible to avoid false positives. Therefore, in order to make the system more stable while working on a video stream acquired from a surveillance camera, we decided to also explicitly exploit the temporal information, by adding a further layer that performs spatial and temporal analysis of the patches (see Figure 7).

This analysis is performed in two steps:

- Frame-based evaluation: A frame is considered positive if it contains at least one patch classified as smoke. This can be considered a kind of spatial aggregation.
- Event-based evaluation: A smoke event is generated if in a time window composed of *k* consecutive frames, at least x% of them are classified as smoke.

Note that in our experiments, we consider a video as smoke if at least an event is generated. We will refer to this as video-based classification.

## 3. Dataset

As mentioned in the introduction, one of the contributions of this paper is a new dataset for smoke detection in videos, namely the MIVIA Smoke Detection Dataset. It is one of the datasets with pixel-level groundtruth, so it can be used also for smoke segmentation.

MIVIA-SDD is designed to benchmark both smoke and fire detectors and has been acquired in the wild: a camera has been mounted in the MIVIA Lab of the University of Salerno, Italy, framing the mountain in front of the lab. Totally, the dataset is composed of 129 videos, with 28 h of footage and a resolution of 292×240 pixels; each video is about 15 min long and contains 7500 frames on average. MIVIA-SDD contains both positive videos, i.e., those with smoke and fire, and negative videos, in which there are only background elements without smoke or fire.

**Figure 8:**
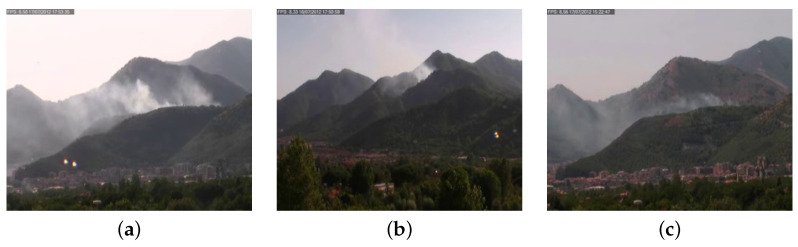
Examples of positive videos from the MIVIA-SDD, with smoke on the mountains with more light (**a**) and less light (**b**,**c**).

**Figure 9:**
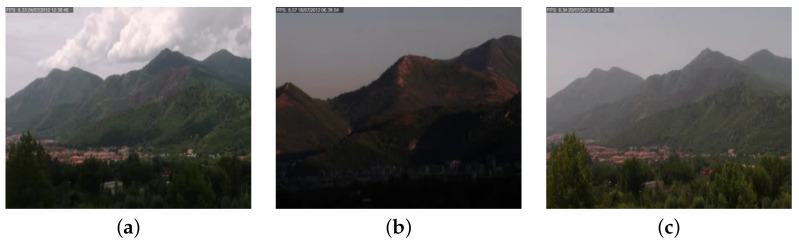
Examples of negative videos from the MIVIA-SDD, without fire in progress, but with clouds (**a**), red shades (**b**) and red houses (**c**).

Table 3 contains details about the number of videos in the dataset and its partition in training, validation and test sets. All the negative video samples are in the test set to evaluate the false positive rate of the proposed system. The dataset has been realized considering environmental elements that can be easily confused as smoke or fire together with challenging environmental conditions, such as red houses in a wide valley, mountains at sunset, sun reflections in the camera and clouds moving in the sky. Some positive video examples of the MIVIA-SDD are reported in Figure 8, while negative examples are reported in Figure 9. The dataset is made publicly available in our website (https://mivia.unisa.it/datasets/video-analysis-datasets/smoke-detection-dataset/ accessed on 1 July 2024).

**Figure 10:**
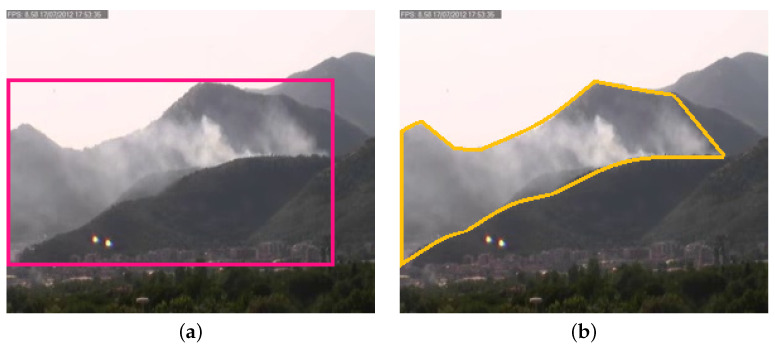
Comparison between bounding box (**a**) and polygon labeling (**b**). The image on the left shows an example of bounding box while the one on the right depicts a polygon annotation example. It is clear that polygon labeling is more appropriate and can better capture the smoke shape without including non-smoke areas.

Concerning the ground truth, a common approach is to provide it in terms of labeled videos (by assigning a label smoke or non-smoke to the whole video), frames (by assigning a label smoke or non-smoke to the single frame) or bounding boxes (by drawing the minimum bounding box around the smoke, frame by frame). Furthermore, as shown in the example in Figure 10, the bounding box for its nature can not be considered representative of the smoke, since it also contains a huge amount of non-smoke pixels. Even if this is a quite common approach for detection problems, it is not the best possible approach when dealing with smoke, given its specific shape. For this reason, we have manually annotated one frame per second by drawing the polygon around the smoke, then the intermediate frames have been automatically annotated through interpolation. It is important to note that such kind of annotation allows for use of the dataset for training both smoke detectors and CNNs for smoke segmentation.

## 4. Experimental Analysis

In this section, we present the experimental protocol and results. Benchmarking the proposed multi-stage system only on the base of its outcome on videos does not allow us to properly evaluate the performance of all the stages involved in the classification process. Therefore, the adopted protocol allows us to take into account separately the capability of a fully deep learning-based approach and the improvement obtained by the combination with the color/motion-based approach. In detail, we have considered three aspects, performing specific experiments for each of them:

- Patch-based: the aim of the analysis at this level is to evaluate the accuracy of the considered CNNs as patch-wise classifiers. All the models have been trained on a dataset of patches randomly extracted from the videos belonging to the training set and tested on those extracted from the test set.
- Frame-based: a frame-by-frame analysis on frames extracted from test set videos allows us to evaluate the entire pipeline discussed in Section 2, consisting of movement evaluation, color-based filtering and patch classification (frame classification).
- Video-based: this last level evaluates the pipeline with the addition of the spatial and temporal analysis (video classification) when classifying the entire video.

### 4.1. Performance Metrics

For all the three kinds of analysis (patch-, frame- and video-based) we consider three performance metrics, namely: accuracy (A) (see Equation (10)), recognition rate (RR) (see Equation (11)) and false positive rate (FPR) (see Equation (12)).

(10) $$A=\frac{\mathrm{TP}+\mathrm{TN}}{\mathrm{TP}+\mathrm{FN}+\mathrm{TN}+\mathrm{FP}}$$

(11) $$\mathrm{RR}=\frac{\mathrm{TP}}{\mathrm{TP}+\mathrm{FN}}$$

(12) $$\mathrm{FPR}=\frac{\mathrm{FP}}{\mathrm{FP}+\mathrm{TN}}$$

All the three metrics are based on the following measures: true positives (TPs) is the number of correct smoke classifications, false negatives (FNs) is the number of smoke samples wrongly classified as background, false positives (FPs) is the number of negative samples wrongly classified as smoke and true negatives (TNs) is the number of correctly classified background samples.

Furthermore, for the video-based evaluation we also compute other two metrics:

- False Positive Videos (FPVs): number of negative videos where at least one temporal window is classified as smoke.
- False Positive Events (FPEs): number of false positive events raised in the negative videos (i.e., the number of temporal windows classified as smoke).

### 4.2. Experimental Results

As introduced in Section 2, we have considered different widely used CNN architectures: MobileNet, VGG-19, ResNet-50, Inception v3 and Xception. We chose these neural networks since they cover most of the architectures widely used in recent years.

The networks are pre-trained on ImageNet, a very large dateset for object detection tasks; then, they have been fine-tuned to classify smoke patches with transfer learning by using the 6,406,047 patches extracted from the videos of the training set and validated on 1,348,275 patches of the validation set. As for the learning procedure, since the task is a binary classification we have considered a binary cross-entropy loss function; the training of the CNNs has been performed using RMSprop with an initial learning rate of $10^{-7}$, a batch size of 128 and an early stopping mechanism that aborts the training if the accuracy on the validation set does not improve for 5 epochs. Finally, the model has been tested over the 4,551,303 patches extracted from the test set videos.

In Table 4, we show the results of the three analyses; in particular accuracy, recognition rate and false positive rate are reported for patch-based and frame-based analyses, while false positive videos and false positive events are used for the video-based one.

We can note that Inception v3 is the model obtaining the best overall accuracy for the patch-based evaluation (89.87%), namely the best trade-off between recognition rate (94.77%) and false positive rate (12.59%). VGG-19 (96.24%) and Xception (95.29%) reach higher recognition rates, but at the cost of higher false positive rates (18.69% and 14.34%). MobileNet and ResNet-50 achieve the worst results both in terms of recognition rate and false positive rate.

For the frame-based evaluation, the results are substantially more balanced, probably due to the fact that the adoption of movement evaluation and color-based filtering allows for selecting the most promising smoke regions. We can note, for this reason, a reduction in both the recognition and the false positive rates. This high sensitivity implies a lower average accuracy and a lower FPR with respect to patch-based analysis. Inception v3 is still the CNN obtaining the best false positive rate (8.61%), paying with the worst recognition rate (73.32%) but retaining the top-2 accuracy (85.55%). In this case, Xception achieves the best accuracy (86.11%), namely the top-1 compromise between recognition rate (77.90%) and false positive rate (9,96%). ResNet-50, VGG-19 and MobileNet achieve very similar results (85.46% vs. 85.45% vs. 85.40% accuracy).

For the video-based evaluation, which is in principle the main analysis of our experimentation, the recognition rate and the false positive rate are higher on average. This is due to the fact that the time persistence of a single patch can trigger a smoke alarm, even if the negative effects are limited by the movement evaluation and the color-based filtering. Inception v3 confirms its superiority against the other networks in terms of false positive rate (14.28%), which in turn corresponds to the highest accuracy (88.68%) with the same recognition rate of Xception, ResNet-50 and VGG-19 (94.44%). In addition, the lowest false positive videos (10 in total, 3 less than Xception, ResNet-50 and VGG-19 and 5 less than Xception) and false positive events (192 in total, 14 less than the top-2 result) confirm the higher effectiveness of Inception v3 with respect to the other networks for video-based smoke detection.

**Figure 11:**
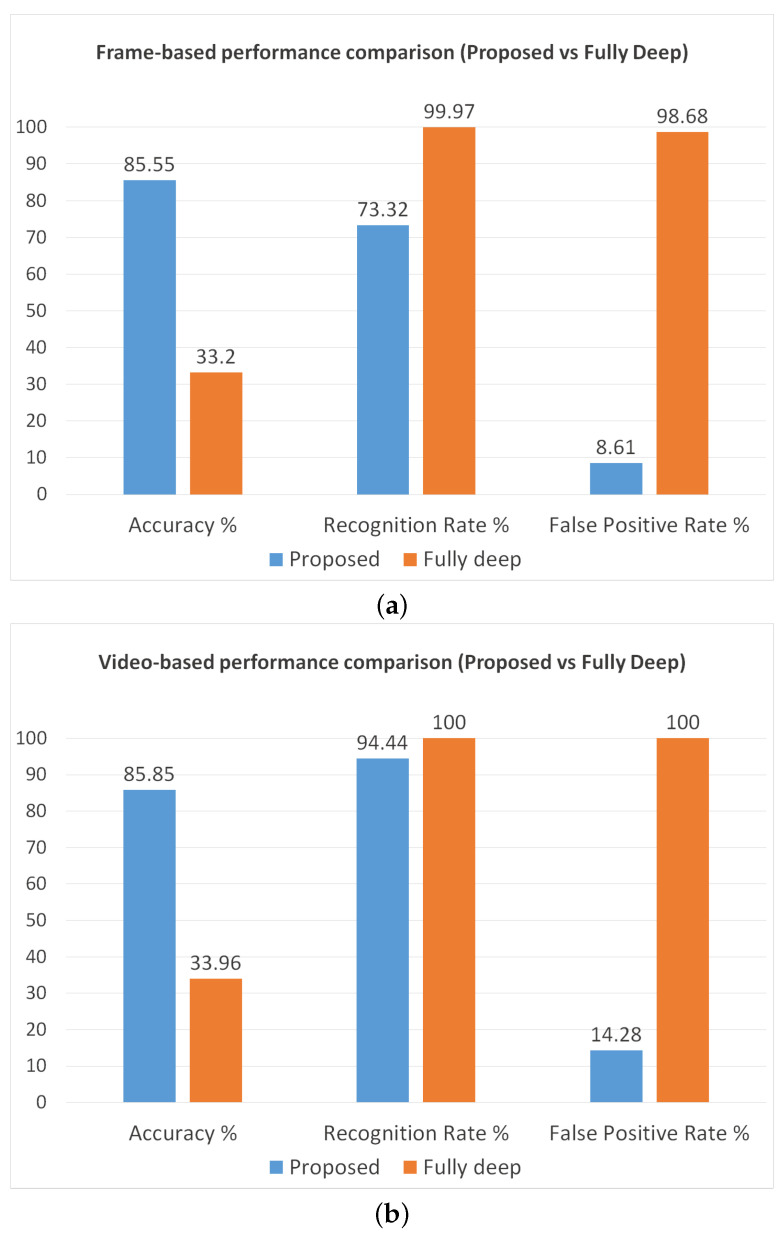
Performance comparison between our proposed method and the fully deep approach. The chart on the top (**a**) shows the frame-based performance, while the other (**b**) represents the video-based performance.

Furthermore, we also perform another analysis to observe the reduction in the false positive rate due to the application of movement evaluation and color-based filtering. As reference we use Inception v3, since it demonstrated the best performance in the previous experiments. The achieved results are reported in Figure 11 and confirm our hypotheses. Indeed, we can note that the system based on the fully deep approach obtains a very high recognition rate (99.97% frame-based, 100% video-based), but in turn also the false positive rate becomes much higher (98.68% frame-based, 100% video-based), making the system unusable in real-world conditions.

This result is quite unexpected by considering the high patch-based accuracy (89.87%); however, the capability of the network to recognize the patches does not imply a remarkable frame-based and video-based performance. In principle, around 10% of the patches may be always misclassified by the CNN, so obtaining very high frame-based and video-based FPR. Therefore, such an experimental result confirms that the movement evaluation and the color-based filtering are essential steps for our method.

**Figure 12:**
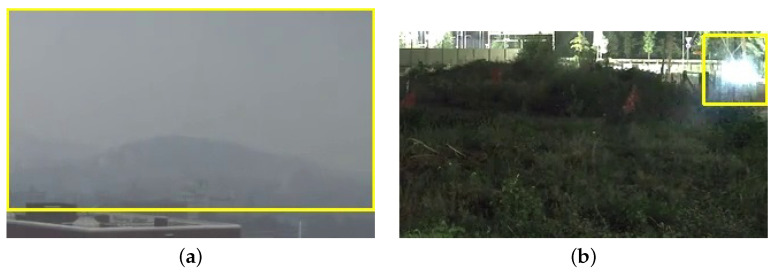
Examples of false positives detected by the algorithm on smoke-like objects, namely fog (**a**) and headlights (**b**).

In conclusion, considering the not negligible number of false positive videos and events, it is worth investigating the causes. After a qualitative analysis, we notice that there are some specific negative videos that are more problematic in terms of false alarms. In fact, MobileNet, VGG-19 and ResNet-50 generate false positive events in the same 13 videos; some examples are reported in Figure 12. The majority of false alarms are raised due to the moving clouds in the sky (especially when they are grayish, otherwise they are filtered out by the color rule) and due to brightness changes in the videos and sudden reflections of the sun on the mountains (e.g., when a cloud goes away the sun suddenly lights up the scene and these changes are detected by the movement evaluation). This evidence suggests that, even combining movement and color rules with deep learning techniques, we are not able to cut down the false positive rate below a certain threshold.

## 5. Conclusions

In this work, we proposed a novel method for video-based smoke detection. It is a hybrid approach combining traditional image processing techniques like movement evaluation and color-based filtering with modern CNNs for smoke recognition. In addition, we proposed a novel challenging dataset, MIVIA-SDD, which was realized by collecting 129 videos in the wild, for 28 h in total; we used it to assess and evaluate the performance of the proposed method. The experimental results show that our system achieves a remarkable recognition rate of more than 94%, keeping the false positive rate lower than the fully deep approach (14% vs. 100%). However, the experimental analysis points out that, despite how we adopted various countermeasures available in the state of the art, there is still room for improving the capability to reduce the false positive rate.

Furthermore, by making our smoke detection dataset publicly available, we hope to promote future research in this field.

## Figures and Tables

**Table 1:** Comparison of our proposed dataset (MIVIA-SDD) with the other datasets present in the literature. The main advantage of our dataset is the larger number of smoke videos and the longer total duration. Note that the total number of videos for Bilkent, FIRESENSE and CVPR KMU datasets also include fire videos (14, 11 and 22 videos, respectively).

Dataset	# Videos	Videos Duration (s)	# Smoke Videos	Smoke Videos Duration (s)
Proposed	129	100,062	59	43,869
Bilkent [35]	38	3143	21	2686
FIRESENSE [37]	49	1753	13	531
SKLFS USTC [14]	3023	1513	2018	1255
CVPR KMU [36]	38	4462	6	731

**Table 2:** Values of the parameters used in our experiments, empirically chosen after a comprehensive grid search on the validation set. A brief description of the parameters is reported, while more details can be found in the specific sections.

Parameter	Value	Section	Description
*T*	120	Section 2.1	Regulates the background update speed
$\tau_{F}$	20	Section 2.1	Foreground threshold
$\tau_{C}$	27	Section 2.2	RGB color difference threshold
$\tau_{S}$	32	Section 2.2	HSV color saturation threshold
$\tau_{V}$	190	Section 2.2	HSV color value threshold
*K*	32	Section 2.3	Side of the patch
$\tau_{NZ}$	0.07	Section 2.3	Smoke patch evaluation threshold
$X_{1}$	250	Section 2.4	Number of frames in the temporal window
*X*	50	Section 2.4	Minimum percentage of smoke frames to raise an alarm

**Table 3:** Training, validation and test set of the MIVIA Smoke Detection Dataset.

Video Type	Training Set	Validation Set	Test Set	Total
Smoke	18	5	36	59
No smoke	0	0	70	70

**Table 4:** Results obtained on the MIVIA-SDD test set in terms of accuracy (A), recognition rate (RR), false positive rate (FPR), false positive videos (FPVs) and false positive events (FPEs). The best results are highlighted in bold.

Method	Patch-Based			Frame-Based			Video-Based				
	A	RR	FPR	A	RR	FPR	A	RR	FPR	FPV	FPE
MobileNet	86.93	94.57	16.89	85.40	74,95	9.61	83.96	88.88	18.57	13	206
VGG-19	86.29	**96.24**	18.69	85.45	75,13	9.62	85.85	**94.44**	18.57	13	211
ResNet-50	86.22	94.38	17.85	85.46	75.09	9.58	85.85	**94.44**	18.57	13	211
Inception v3	**89.87**	94.77	**12.59**	85.55	73.32	**8.61**	**88.68**	**94.44**	**14.28**	**10**	**192**
Xception	88.87	95.29	14.34	**86.11**	**77.90**	9.96	83.96	**94.44**	21.42	15	218

## Data Availability

The dataset is available under request at the following link: https://mivia.unisa.it/datasets/video-analysis-datasets/smoke-detection-dataset/ accessed on 1 July 2024.
